# Action of Neurotransmitter: A Key to Unlock the AgRP Neuron Feeding Circuit

**DOI:** 10.3389/fnins.2012.00200

**Published:** 2013-01-21

**Authors:** Tiemin Liu, Qian Wang, Eric D. Berglund, Qingchun Tong

**Affiliations:** ^1^Division of Hypothalamic Research, Department of Internal Medicine, The University of Texas Southwestern Medical Center at DallasDallas, TX, USA; ^2^The Brown Foundation Institute of Molecular Medicine, The University of Texas Health Science Center at HoustonHouston, TX, USA

**Keywords:** AgRP neurons, parabrachial nucleus, paraventricular hypothalamus, feeding

## Abstract

The current obesity epidemic and lack of efficient therapeutics demand a clear understanding of the mechanism underlying body weight regulation. Despite intensive research focus on obesity pathogenesis, an effective therapeutic strategy to treat and cure obesity is still lacking. Exciting studies in last decades have established the importance of hypothalamic agouti-related protein-expressing neurons (AgRP neurons) in the regulation of body weight homeostasis. AgRP neurons are both required and sufficient for feeding regulation. The activity of AgRP neurons is intricately regulated by nutritional hormones as well as synaptic inputs from upstream neurons. Changes in AgRP neuron activity lead to alterations in the release of mediators, including neuropeptides Neuropeptide Y (NPY) and AgRP, and fast-acting neurotransmitter GABA. Recent studies based on mouse genetics, novel optogenetics, and designer receptor exclusively activated by designer drugs have identified a critical role for GABA release from AgRP neurons in the parabrachial nucleus and paraventricular hypothalamus in feeding control. This review will summarize recent findings about AgRP neuron-mediated control of feeding circuits with a focus on the role of neurotransmitters. Given the limited knowledge on feeding regulation, understanding the action of neurotransmitters may be a key to unlock neurocircuitry that governs feeding.

## Introduction

The ability to harness energy from a variety of metabolic pathways is a property of all living organisms. For a multi-organ system like rodents and mammals, different organs have evolved to perform distinct functions to maintain energy balance. For example, the brain controls the intake of energy (mostly carbohydrates, fats, and proteins), which is absorbed by the digestive system, trafficked by the liver, and distributed to the body via the circulation system (Schwartz et al., 2000; Saper et al., 2002; Elmquist and Flier, 2004). A disturbance in the delicate balance between energy intake and energy requirements in the body will lead to changes in metabolism and body growth (body weight). During evolution when food is not always available, excess energy is stored as fat, which can be used when food is scarce. Thus, for a given living subject, energy balance is depicted as energy intake = internal heat produced + external work + energy storage (fat; Saper et al., 2002). Obesity is defined as the excessive fat accumulation. The current obesity epidemic and lack of efficient therapeutics demand a clear understanding of the mechanisms underlying body weight regulation. It is now well established that the brain, especially the hypothalamus, maintains body weight homeostasis by effectively adjusting food intake and energy expenditure (internal heat production and external work) in response to changes in the levels of various nutritional status indicators such as leptin, insulin, ghrelin, and others (Elmquist et al., 1999; Pinto et al., 2004; Nogueiras et al., 2008; Friedman, 2009; Yang et al., 2011; Heppner et al., 2012; Liu et al., 2012). Recent studies have identified important groups of neurons and genes in the hypothalamus for energy balance regulation (Cone, 2005; Elmquist et al., 2005).

In regard to feeding control, emerging results demonstrate orexigenic Agouti-related protein (AgRP) neurons in the arcuate nucleus (Arc) of the hypothalamus are critical regulators of feeding and food-seeking behavior (Ollmann et al., 1997; Cone, 2005; Flier, 2006). Activation of AgRP neurons in mice causes hyperphagia, increases motivation to work for food, and initiates intense food-seeking behavior (Aponte et al., 2011; Krashes et al., 2011), while inhibition of AgRP activity or ablation of AgRP neurons leads to reduced feeding or starvation (Bewick et al., 2005; Gropp et al., 2005; Luquet et al., 2005; Xu et al., 2005a; Wu et al., 2009). Since the activity level of AgRP neurons is a determinant for feeding, it will be important to understand how the activity of AgRP neurons is controlled. The ultimate result of changes in AgRP neuron activity is alterations in neurotransmitter release, which is the only way for neurons to transmit signals to downstream targets. Deciphering the function of neurotransmitters released from AgRP neurons and their downstream targets represents a critical step toward understanding the AgRP neural pathway for feeding control. This review discusses recent findings on the control the AgRP activity and the function of neurotransmitters from AgRP neurons.

## Upstream Regulators of AgRP Neurons

### Upstream humoral regulators of AgRP neurons – leptin, ghrelin, insulin

**Figure 1:**
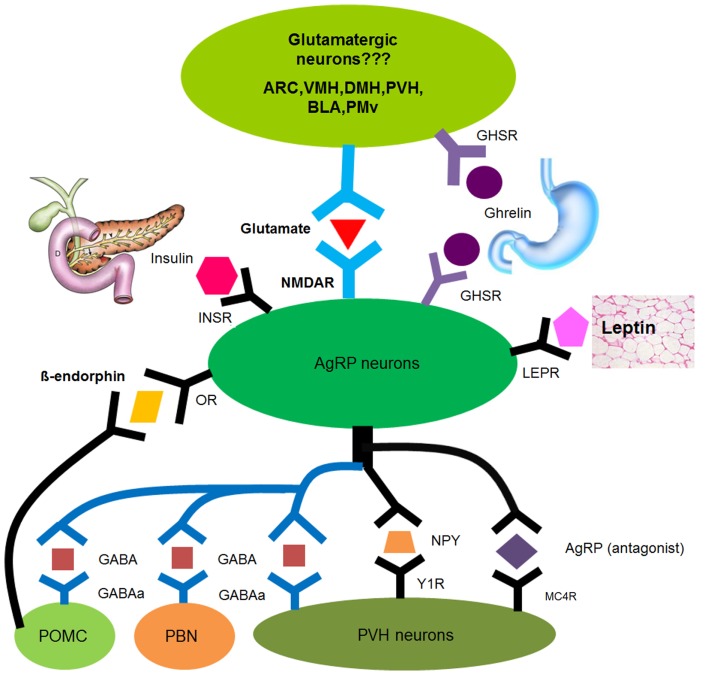
**Model underlying the AgRP Neuron Feeding Circuit**. A summary about upstream and downstream mediators of AgRP neurons including (1) neuronal regulators-glutamate, GABA, AgRP, NPY, and β-endorphin; (2) humoral regulators – leptin, ghrelin, insulin.

Agouti-related protein neurons in the hypothalamic Arc are crucial targets of feeding hormones. One of these hormones is ghrelin, which promotes positive energy balance (Tschop et al., 2000; Nakazato et al., 2001; Nogueiras et al., 2008). Ghrelin was first identified in 1999 as an endogenous ligand of the growth hormone secretagogue receptor (GHSR) and is synthesized and secreted mainly from endocrine cells of the stomach and intestine (Kojima et al., 1999; Wierup et al., 2007). Both central and peripheral administrations of ghrelin stimulate appetite and food intake, increase body weight, and promote adiposity and decrease energy expenditure in rodents indicating an orexigenic impact via central signaling (Tschop et al., 2000; Wren et al., 2000; Asakawa et al., 2001; Nakazato et al., 2001). Histological examination of GHSR showed its presence in several hypothalamic nuclei including Arc and paraventricular nucleus of the hypothalamus (PVH) and direct binding of ghrelin in these hypothalamic regions was also found (Cowley et al., 2003; Zigman et al., 2006). In these potential sites of ghrelin action, the Arc where GHSR mRNA is abundantly expressed, is thought to contain the primary ghrelin-responsive neurons mediating effects on feeding and body weight. GHSRs are predominantly expressed in the orexigenic cell population in the Arc, AgRP neurons (Figure 1), in contrast to few GHSRs in proopiomelanocortin (POMC) neurons, the anorexigenic population in the Arc (Willesen et al., 1999; Nogueiras et al., 2008). The neuronal activity of AgRP neurons is triggered by ghrelin indicated by increased electrical activity and c-fos immunoreactivity (Cowley et al., 2003; Andrews et al., 2008). Therefore, ghrelin is suggested to inhibit POMC neurons indirectly by activating AgRP neurons (Cowley et al., 2003; Tong et al., 2008; Wu et al., 2008a; Atasoy et al., 2012). Feeding stimulation by ghrelin is abolished in AgRP/Neuropeptide Y (NPY) double knockout mice and ablation of AgRP neurons in adulthood indicating that ghrelin signaling in AgRP neurons is important for controlling feeding (Chen et al., 2004; Bewick et al., 2005).

Leptin, which counter-acts the effects of ghrelin to regulate energy balance and food intake, is a hormone produced by fat tissue. The gene for leptin was first cloned in 1994 (Zhang et al., 1994) followed by the gene for the leptin receptor (LEPR) in 1995 (Tartaglia et al., 1995). Mice with leptin deficiency (*ob/ob*) or deficits in its receptor (*db/db*) show severe obesity phenotype thus supporting a crucial role for leptin signaling to regulate feeding and energy expenditure (Friedman, 1998; Friedman and Halaas, 1998). LEPR mRNA is highly expressed within the hypothalamus including the ventromedial nucleus of the hypothalamus (VMH), the dorsomedial nucleus of the hypothalamus (DMH), and the Arc where LEPR is densely expressed in both AgRP and POMC neurons (Figure 1; Mercer et al., 1996a,b; Elmquist et al., 1998, 1999; Elias et al., 1999). Indeed, direct leptin action on AgRP neurons has been revealed by the obesity phenotype caused by specific deletion of LEPR in AgRP neurons (van de Wall et al., 2008). Activation of leptin-responsive neurons involves induction of the JAK-STAT3 signaling pathway, which regulates the gene expression (Bates et al., 2003; Bates and Myers, 2004). Consistent with its anorexigenic role, leptin administration directly inhibits neuronal activity of AgRP neurons and depolarizes POMC neurons (Spanswick et al., 1997; Cowley et al., 2001; Elmquist et al., 2005). The direct but opposite actions of leptin in AgRP and POMC neurons are further confirmed by (1) induction of STAT3 in both populations; (2) increased c-fos immunoreactivity in POMC neurons, but not in AgRP neurons after leptin treatment (Elias et al., 1999). Although GHSR and LEPR are co-expressed in the medial part of the Arc, the function of LEPR is likely to be independent of GHSR indicated by the evidence that anorectic effects of exogenously administrated leptin were similar in wild-type and GHSR knockout mice (Perello et al., 2012).

Insulin is a hormone made and secreted by beta cells in the pancreas and plays a pivotal role in regulating blood glucose. An elevation of blood glucose stimulates pancreatic beta cells to secrete insulin. The increase in circulating insulin leads to accelerated glucose uptake into peripheral tissues, which is essential to maintain glucose homeostasis (Shepherd and Kahn, 1999). In addition to well known gluco-regulatory actions of insulin on peripheral tissues, insulin signaling in the brain (especially the hypothalamus) also critically regulates blood glucose (Obici et al., 2002; Plum et al., 2006; Paranjape et al., 2010; Levin and Sherwin, 2011). Insulin receptors (INSR) are expressed in many CNS regions including the hypothalamus (Figure 1; Werther et al., 1987; Unger et al., 1991). Among these hypothalamic neurons, insulin/PI3K signaling in both AgRP and POMC neurons is required for maintaining glucose homeostasis (Konner et al., 2007; Hill et al., 2010; Lin et al., 2010). As early as 1979, intracerebroventricular infusion of insulin was shown to reduce body weight and food intake in baboons, a feature of anorexigenic hormone (Woods et al., 1979). Recent studies suggest that insulin acts on AgRP neurons to exert this anorexigenic role, which is mediated by activation of phosphatidylinositol 3-kinases (PI3K) and the nuclear export of the forkhead transcription factor (Foxo1; Kim et al., 2006; Ren et al., 2012). Like leptin, insulin hyperpolarizes AgRP neurons, which is consistent with its role to reduce food intake and body weight (Spanswick et al., 2000; Konner et al., 2007). Although LEPR and INSR co-express in both AgRP and POMC cell populations, the actions of LEPR and INSR are integrated by PI3K in POMC neurons, but not in AgRP neurons (Xu et al., 2005b).

### Upstream neuronal regulators of AgRP neurons – glutamate

Agouti-related protein neurons are regulated by numerous humoral and neural inputs. While intense focus has been placed on identifying and understanding humoral inputs to AgRP neurons (e.g., leptin, ghrelin, insulin; Elmquist et al., 1999; Belgardt et al., 2009; Friedman, 2009; Castaneda et al., 2010; Perello and Zigman, 2012), very little is known about neural regulation of AgRP neurons. Recent findings that neurotransmitter inputs to AgRP neurons (e.g., glutamate and GABA) regulate food intake suggest that abnormal eating behaviors and dramatic weight change may be linked to disturbances in neural regulation of AgRP neurons (Pinto et al., 2004; Yang et al., 2011; Liu et al., 2012).

In a series of elegant *in vitro* electrophysiological, pharmacogenetic, and optogenetic experiments on acute brain slices in fed and food-deprived mice, Yang et al. (2011) found that fasting activation of AgRP neurons doubled the frequency of excitatory postsynaptic currents, but not amplitude. They also investigated in great detail the paired-pulse ratio (PPR) in wild-type mice and concluded that the very low PPR of glutamatergic inputs onto AgRP neurons in *ad libitum* fed mice limits the capability to observe any further reduction of PPR after food deprivation. Consistent with this, they showed a dissociation of PPR in fed and fasted mice upon lowering extracellular calcium (a common method for revealing changes in presynaptic release properties of high release probability synapses). Together, these lines of evidence suggest a ghrelin/ghrelin receptor/AMP-activated protein kinase pathway operating in presynaptic glutamatergic neurons (Figure 1).

Other potential mechanisms have also been suggested. Pinto and colleagues measured EPSCs on AgRP neurons (via *in vitro* electrophysiological recordings) and analyzed excitatory synapses on perikarya (using electron microscopic stereology) and provided data suggesting a postsynaptic mechanism. Specifically, they found that increased frequency of EPSCs on NPY/AgRP neurons in leptin-deficient *ob/ob* mice was associated with increased excitatory synapses on the perikarya of NPY/AgRP neurons (Pinto et al., 2004).

There is additional support for a postsynaptic mechanism. A recent study by Liu et al. (2012) evaluated the role of glutamatergic input to AgRP neurons and excitatory synapses on the dendritic spines of AgRP neurons. Their strategy was to decrease glutamate input to AgRP neurons by selectively deleting the ionotropic glutamate receptor, NMDARs (Malenka and Nicoll, 1999; Kessels and Malinow, 2009; Collingridge et al., 2010). Using Cre-loxP technology, the authors crossed *Agrp*-ires-Cre mice (Tong et al., 2008) with lox-NR1 mice (Tsien et al., 1996; NR1 is a required subunit of the NMDA receptor, deletion of NR1 subunits causes total loss of NMDAR activity) to generate *Agrp*-ires-Cre, NR1^lox/lox^ mice (Voglis and Tavernarakis, 2006; Higley and Sabatini, 2008; Bito, 2010). They found that decreased glutamate input to AgRP neurons was associated with decreased body weight, food intake, rates of re-feeding, EPSC frequency, and dendritic spine number. Importantly, fasting did not alter the PPR in wild-type mice. Liu and colleagues suggested that postsynaptic NMDARs are required for the fasting-induced increase in EPSC frequency that is paralleled by dendritic spinogenesis. They propose a tentative model to explain how fasting regulates the activation of AgRP neurons: fasting → dendritic spinogenesis → formation of new excitatory synapses → increased glutamatergic transmission → activation of AgRP neurons.

However, the findings of Liu et al. could not rule out the possibility of a presynaptic mechanism, suggesting that presynaptic and/or postsynaptic mechanisms may apply concurrently or assume dominance depending on nutritional status.

The obvious question is to identify the source(s) of glutamatergic input to AgRP neurons. Candidates will be glutamatergic neurons expressing ghrelin receptors (Atasoy et al., 2012) including the Arc, VMH, DMH, PVH, basolateral nucleus of the amygdala (BLA), and ventral premammillary nucleus (PMv; Figure 1; Zigman et al., 2006; Johnson et al., 2009; Vong et al., 2011; Perello et al., 2012). One possible method is to use a monosynaptic rabies approach to determine the source(s) of presynaptic input to AgRP neurons. Rabies virus, a neuronal circuit tracer in the retrograde direction, can be used to target the monosynaptic input to AgRP neurons (Marshel et al., 2010; Wall et al., 2010). Compared to standard electrophysiological techniques, this approach will reveal direct monosynaptic inputs to AgRP neurons by controlling the initial rabies virus infection and subsequent monosynaptic retrograde spread. These studies should identify glutamatergic neurons directly projecting to AgRP neurons to drive feeding behavior.

## Downstream Mediators of AgRP Neurons

Agouti-related protein neurons directly sense changes in nutritional status hormones such as leptin, insulin, ghrelin, and others, and neurotransmitters such as glutamate, GABA, and others to modulate energy homeostasis. As a result, AgRP neuron activity dramatically increases in response to fasting, signaling a need to eat (Takahashi and Cone, 2005). AgRP neurons also release downstream effectors (neural-GABA, AgRP, and NPY) to drive feeding (Figure 1). Interestingly, compared to controls, increasing the activity level of AgRP neurons induces hyperphagia whereas reducing the activity level of those neurons induces hypophagia (Krashes et al., 2011), suggesting that the activity level of AgRP neurons correlates with the level of feeding. These data suggest that AgRP neurons are both sufficient and required for feeding regulation.

### Downstream neuropeptide mediators of AgRP neurons – AgRP and NPY

Neuropeptide Y and AgRP are two neuropeptides released from AgRP neurons that potently induce feeding, consistent with the role of AgRP neurons to promote feeding (Elmquist et al., 2005). Ghrelin increases feeding behavior by stimulating production of NPY and AgRP in AgRP neurons whereas leptin and insulin inhibits food intake and body weight by decreasing their expression (Schwartz et al., 1992; Korner et al., 2001; Zigman and Elmquist, 2003; Chen et al., 2004; Morrison et al., 2005; Goto et al., 2006).

Ectopic expression of agouti protein in mice (*Ay/a*) causes an obese phenotype (Bultman et al., 1992). AgRP, a homology of Agouti, was identified to be up-regulated in obese mice suggesting a potential role of AgRP in feeding and energy balance regulation (Shutter et al., 1997). Introcerebroventricular injection of AgRP or its expression in a transgenic mouse model both lead to hyperphagia, lowered energy expenditure, and obesity (Graham et al., 1997; Rossi et al., 1998; Small et al., 2001). The effect of a single injection of AgRP to promote feeding is long-lasting, which is different from other orexigenic hormones including ghrelin and NPY (Schwartz et al., 2000; Hagan et al., 2001). AgRP affects feeding behavior and metabolism by antagonizing the melanocortin receptors, MC3R and MC4R, which are stimulated by POMC cleavage products (Fan et al., 1997; Ollmann et al., 1997). Besides the mechanism of competitive antagonism, AgRP is also suggested to regulate feeding behavior and energy balance as an inverse agonist of the central melanocortin system (Haskell-Luevano and Monck, 2001; Nijenhuis et al., 2001; Tolle and Low, 2008). Distinctive from other modulators mentioned above, NPY is the only one widely expressed throughout the body, both the CNS and PNS in mammals (Lundberg et al., 1982; Tatemoto et al., 1982). It is present in many brain regions including the hippocampus, hypothalamus, amygdala, cortex (Gray and Morley, 1986; Wahlestedt et al., 1989), and the peripheral nervous system including sympathetic post-ganglionic neurons and the adrenal medulla, and peripheral organs such as the pancreas and spleen (Lundberg et al., 1982; Ericsson et al., 1987; Klimaschewski et al., 1996; Whim, 2006, 2011). The known NPY receptors include Y1, Y2, Y4, and Y5 as well as Y6 in the mouse. All Y receptors are G protein coupled receptors and their activation usually causes inhibitory responses such as inhibition of cAMP accumulation (Michel et al., 1998).

In the CNS, NPY is most highly co-expressed with AgRP in the Arc and substantial amounts of NPY are also found in other hypothalamic nuclei including the dorsomedial nucleus, and numerou NPYergic neurons project into the PVH (Chronwall et al., 1985; van den Pol et al., 2009). NPY was first found to promote hyperphagia based on evidence that intraventricular administration of this peptide significantly induced feeding behavior in rats (Clark et al., 1984, 1985; Levine and Morley, 1984; Stanley and Leibowitz, 1984). Moreover, central administration of NPY reduces energy expenditure and chronic infusion of NPY can induce obesity due to overeating (Billington et al., 1991; Flier and Maratos-Flier, 1998).

Surprisingly, NPY knockout mice on a 129/SvCp-J background did not show a phenotype of reduced feeding, body weight, or adiposity under normal conditions (Erickson et al., 1996a). However, genetic removal of NPY attenuated hyperphagia and obesity phenotype in leptin-deficient mice (Erickson et al., 1996b). In addition, when NPY knockout mice were backcrossed onto a C57BL/6 background, they showed decreased re-feeding after fasting (Bannon et al., 2000). This is similar to Y1 knockout mice that display slightly diminished feeding and strongly reduced fasting-induced re-feeding (Pedrazzini et al., 1998). The Y2 knockout mouse model exhibits decreased body weight gain and deletion of Y2 receptors in the adult mouse hypothalamus led to transiently decreased body weight, but increased food intake indicating a functional role of hypothalamic Y2 receptors to control feeding (Sainsbury et al., 2002a,b). In addition, Y5-deficient mice show normal food intake and body weight, but develop obesity after 30 weeks of age (Marsh et al., 1998). Collectively, this literature studying roles of NPY and NPY receptors in feeding and energy balance supports the importance of central NPY signaling in regulating energy homeostasis.

### Downstream neuronal mediators of AgRP neurons-GABA

As reviewed above, extensive studies have been focused on the role of neuropeptides NPY and AgRP from AgRP neurons. The role of fast-acting neurotransmitter GABA has also been speculated to be important (Horvath et al., 1997; van den Pol, 2003), but was largely neglected until recently. To directly examine the role of GABA release from AgRP neurons, Tong et al. (2008) generated mice with disruption of GABA release specifically from AgRP neurons by inactivating vesicular GABA transporter (VGAT). Tong et al. (2008) found that GABA release from AgRP neurons is required for normal body weight regulation, and disruption of GABA leads to increased energy expenditure and resistance to diet induced obesity. However, the degree in change of body weight due to disruption of GABA release is mild, suggesting that either other neurotransmitters released from AgRP neurons are important or that disruption of GABA release during early embryonic phases invokes developmental compensation which masks physiological effects of GABA release. Consistent with the latter hypothesis, mice with lesions of AgRP neurons in neonates exhibit a mild reduction in body weight while those with lesions of AgRP neurons in adults exhibit significantly reduced food intake or starved to death (Bewick et al., 2005; Gropp et al., 2005; Luquet et al., 2005; Xu et al., 2005a; Wu et al., 2009). It is interesting to point out that mice with lesions of AgRP neurons have been made in several laboratories, and the phenotypes of these mice range from mild reduction, more pronounced reduction in body weight, or starvation-induced death (Bewick et al., 2005; Gropp et al., 2005; Luquet et al., 2005; Xu et al., 2005a). These variant results might be due to different approaches used to lesion AgRP neurons. Lesion of AgRP neurons through inactivating mitochondria might take more time to kill AgRP neurons, allowing for developmental compensation, resulting in mild effects on body weight (Xu et al., 2005a). Lesion of AgRP neurons mediated by diphtheria toxin (DTX) is a rapid process, resulting in more pronounced effects on body weight and feeding (Gropp et al., 2005; Luquet et al., 2005). Nonetheless, these studies demonstrate powerful developmental compensation in response to loss of AgRP neurons in neonatal stages. Mice with AgRP neuron lesions exhibit abundant c-fos expression in known AgRP neuron projection sites including the PVH and parabrachial nucleus (PBN; Wu et al., 2008b). Strong c-fos expression is consistent with loss of GABA projection. However, accompanying with the c-fos expression, there are also abundant gliosis responses in both Arc and AgRP neuron projection sites (Wu et al., 2008b). Whether the gliosis contributes to hypophagia in these mice has not been explored. It would be interesting to directly examine the role of GABA release from AgRP neurons in adult mice through inducible deletion of VGAT. Notably, mild phenotypes by NPY knockout might not be due to developmental compensations since mice with NPY deletion in adults show minimal phenotype in energy balance (Ste Marie et al., 2005).

Given the established importance of AgRP neurons in feeding regulation, intense interests have been generated to look for the responsible neurotransmitters from these neurons. Recent exciting results from Sternson and Palmiter laboratories suggest that GABA is the responsible neurotransmitter and more importantly, they have identified that the neurons in the PVH and PBN, but not POMC neurons, are the direct downstream sites that receive GABA to mediate feeding regulation by AgRP neurons (Wu et al., 2009; Atasoy et al., 2012).

Previous electrophysiological data suggest that AgRP neurons send direct GABAergic projections to nearby POMC neurons (Cowley et al., 2001). Coupled with an important role of the melanocortin system in feeding regulation (Elmquist et al., 2005), these results triggered the speculation that POMC neurons are one of downstream sites to mediate AgPR neuron action on feeding (Cone, 2005). This speculation has not been directly tested until recently. Using a combination of optogenetic and designer receptor exclusively activated by designer drugs (DREADD) approaches, Sternson laboratory elegantly demonstrated that, indeed, AgRP neurons send direct GABAergic projections to POMC neurons; however, surprisingly, POMC neurons play a minimal role in mediating AgRP neurons on acute feeding (Atasoy et al., 2012). Consistently with this finding, results from Palmiter laboratory suggested that starvation resulted from AgRP neurons is independent of the melanocortin pathway (Wu et al., 2009). These data demonstrate that POMC neurons are not a major part of AgRP neuron feeding pathway, at least in acute hyperphagia response. Strikingly, there is no direct projection from POMC neurons to AgRP neurons, or no reciprocal projections between POMC or AgRP neurons, suggesting a rather simple, one way circuit from AgRP to POMC neurons (Atasoy et al., 2012). Whether the physiological significance of GABAergic regulation of POMC neurons by AgRP neuron lies in long term regulation of feeding or in energy expenditure regulation remains to be established. Likewise, the function of potential GABAergic projection from AgRP neurons to non-POMC neurons in the Arc is unknown.

Previous data also suggest the importance of Arc projections to the PVH in energy balance (Elmquist et al., 2005) including feeding and energy expenditure. For example, the melanocortin pathway from POMC neurons to the PVH has been suggested to selectively control feeding (Balthasar et al., 2005). It has also been speculated that GABAergic neurons in the Arc mediate a major part of leptin action on body weight through projections to the PVH (Cone and Simerly, 2011). Recent data from Lowell laboratory demonstrated that direct GABAergic projections from a novel subset of Arc neurons expressing Cre in Rip-Cre mice to the PVH play a selective role in energy expenditure (Kong et al., 2012). It is well accepted that the PVH is one major downstream site of AgRP neurons (Cone, 2005; Elmquist et al., 2005). However, a direct demonstration of a role of AgRP neuron projection to the PVH in feeding regulation is lacking until recently. Based on similar sets of elegant experiments to those for POMC neurons, Sternson laboratory demonstrated a critical role for GABAergic projections from AgRP neurons to the PVH in promoting feeding by AgRP neuron activation (Atasoy et al., 2012). Specifically, by concurrent activation of PVH neurons and AgRP neurons, they showed that activation of PVH neurons effectively reverses hyperphagic effects by AgRP neurons activation (Atasoy et al., 2012), suggesting that AgRP neuron induced hyperphaga is due to a heightened GABAergic tone to the PVH and that suppression of PVH neurons is required for AgRP neuron-mediated hyperphagia. Furthermore, by selectively photostimulating AgRP fibers the PVH, their results showed that suppression of PVH neurons by AgRP neurons is sufficient to mediate AgRP neuron-mediated hyperphagia. Thus, suppression of PVH neurons is both sufficient and necessary for AgRP neuron-mediated hyperphagia. Interestingly, a marked strengthening of GABAergic innervations from AgRP neurons to PVH neurons was found to be associated with NPY deficiency. Given the importance to GABAergic innervations from AgRP neurons to PVH neurons in feeding regulation, this plastic changes in GABAergic action may provide an explanation for lack of physiologic phenotypes by NPY deficiency. Consistently, NPY receptor blockage significantly blunted the hyperphagia effect caused by photostimulation of either AgRP fibers in the PVH or AgRP neurons, suggesting that both GABA and NPY signaling appeared to be necessary for feeding evoked by the GABAergic projection from AgRP neurons to PVH neurons (Atasoy et al., 2012). It would be interesting to identify how NPY deficiency causes changes in GABAergic action.

Given the strong feeding inhibition induced by AgRP neuron inhibition (Aponte et al., 2011; Krashes et al., 2011), it would be interesting to examine whether PVH neurons mediate feeding-reducing effects by AgRP neurons. A direct testing of this has not been reported. However, using a extreme case of AgRP neurons inhibition, i.e., AgRP neuron lesion, Palmiter group found that PVH specific administration of GABA-A agonist failed to rescue the starvation phenotype by AgRP neuron lesion, suggesting that GABAergic projection from AgRP neurons to the PVH has no contribution to the starvation phenotype produced by AgRP neuron lesion (Wu et al., 2009). Of note, the failure in rescuing is not due to insignificant GABAergic input from AgRP neurons since abundant c-fos expression, an indicator of neuronal activation, was observed in the PVH as a result of AgRP neuron lesion, and importantly, the increased c-fos expression were blunted by the application of GABA-A receptor. This data suggest that excitation of PVH neurons contributes minimally to the starvation phenotype by AgRP neurons lesion. Since PVH neuron excitation by GABA-A antagonists suppresses feeding (Kelly et al., 1979), this result suggests that GABAergic action in the PVH from non-AgRP neurons might mediate feeding suppression. In pursuit of downstream targets to GABAergic projections from AgRP neurons that mediate the starvation effects of AgRP neuron lesion, Palmiter group found that the starvation phenotype can be rescued by specific delivery of GABA-A receptor agonist in the PBN (Wu et al., 2009), highlighting an important role for GABAergic projections from AgRP neurons to the PBN. Consistently, adult lesion of AgRP neurons increases PBN neuron activity (Wu et al., 2008b). Taken together, these data suggest a role for a decreased GABAergic tone from AgRP neurons to the PBN in modulating hypophagia response (Figure 1). The PBN is traditionally viewed as a center for taste aversion regulation, which, if activated by sensory inputs from the gut, induces taste aversion, serving as a general protective mechanism to avoid unpleasant food experienced before (Reilly, 1999). Thus, it appears that both hypophagia and taste aversion responses share the same neural pathway. In addition to GABAergic inputs from AgRP neurons, it appears that PBN neurons are also controlled by glutamatergic inputs from the nucleus of solitary tracts, which might mediate inputs from other neurons such as serotoninergic neurons as well as sensory inputs from the gut (Wu et al., 2012). An interesting question is whether this projection also mediates hyperphagic effects produced by increased AgRP neuron activity. Surprisingly, Sternson group found that specific photostimulation of AgRP fibers in the PBN fails to induce the acute hyperphagic effects by AgRP neuron activation (Atasoy et al., 2012), suggesting that acute hyperphagia induced by AgRP neuron activation is not due to heightened GABAergic tone to the PBN.

Caution should be exercised to directly compare the results between Sternson and Palmiter groups since the former deals with rapid changes in neuronal activity with acute feeding and the latter deals with slow alterations in neuronal circuitry with long term feeding effects. Nonetheless, the results from the two groups taken together suggest that GABAergic action from AgRP neurons to the PVH may preferentially mediate hyperphagic effects by AgRP neuron excitation, and that to the PBN may preferentially mediate hypophagic effects by AgRP neuron inhibition. This speculation is in line with the minimal phenotypes observed in mice with AgRP and NPY deficiency (Qian et al., 2002), although the latter can be explained by potential compensatory changes in GABAergic action (Atasoy et al., 2012). It may be the case that GABAergic projection from AgRP neurons to the PVH projection is more engaged during fasting whereas that to the PBN is more engaged during fed states. In addition to the PVH and PBN, AgRP neurons also send GABAergic projections to several other brain sites, and some of these sites might be important to mediate AgRP neuron feeding effects (Atasoy et al., 2012), but still await further demonstration. Further studies stemmed from these results will shed new lights on AgRP neuron feeding circuits.

## Summary

Several laboratories have used a combination of mouse genetics and pharmacology to achieve specific lesion of adult AgRP neurons, state-of-the-art optogenetics, and DREADD (Alexander et al., 2009; Wu et al., 2009; Krashes et al., 2011; Atasoy et al., 2012) to achieve specific manipulation of adult AgRP neurons. Given the complexity of feeding behavior, these studies represent a significant leap in understanding of brain feeding circuits. It appears that AgRP neurons have evolved to be one of major determinants for an individual to harness energy from the environment. With this in mind, modulating the activity of AgRP neurons represents one strategy to control appetite in contexts of the current obesity epidemic (Ren et al., 2012).

It is certain that AgRP neurons are not the only key neurons controlling feeding because lesion of the Arc including AgRP neurons leads to hyperphagia and obesity. Thus, in the Arc, there must be other food intake-inhibiting neurons, lesion of which overrides the action of AgRP neuron lesion. Nonetheless, based on the exciting results focusing on AgRP neurons and using newly developed and highly informative techniques (mouse genetics, optogenetics, and DREADD), future studies will provide more insights on brain circuits controlling food intake.

## Conflict of Interest Statement

The authors declare that the research was conducted in the absence of any commercial or financial relationships that could be construed as a potential conflict of interest.
